# Clinical Significance of the Combination of Serum HE4 Levels, Hemoglobin-to-Red Cell Distribution Width Ratio, and CT Imaging for the Pretreatment Assessment of Adnexal Masses

**DOI:** 10.7150/jca.81174

**Published:** 2023-02-27

**Authors:** Tae-Kyu Jang, Heungyeol Kim, Wankyu Eo, Ki Hyung Kim, Chul Min Lee, Minyoung Kim

**Affiliations:** 1 Department of Obstetrics and Gynecology, Dongsan Medical Center, Keimyung University School of Medicine, Daegu, Republic of Korea.; 2 Department of Obstetrics and Gynecology, Hannah Hospital, Busan, Republic of Korea.; 3 Department of Internal Medicine, College of Medicine, Kyung Hee University, Seoul, Republic of Korea.; 4 Department of Obstetrics and Gynecology, Pusan National University School of Medicine, Biomedical Research Institute and Pusan Cancer Center, Pusan National University Hospital, Republic of Korea.; 5 Department of Obstetrics and Gynecology, Cha University, Ilsan Medical Center School of Medicine, Seoul, Republic of Korea.; 6 Department of Nursing, Kyungnam College of Information and Technology, Busan, Republic of Korea.

**Keywords:** HE4 protein, Humans, Red cell distribution width, Computed tomography, Spiral, Adnexal disease.

## Abstract

**Background**: This study aimed to determine the optimal combination of biomarkers that can predict epithelial ovarian cancer (EOC) and compare the combination with the risk of ovarian malignancy algorithm (ROMA) or Copenhagen index (CPH-I).

**Methods**: Data from 66 patients with EOC and 599 patients with benign ovarian masses who underwent definitive tissue diagnosis of adnexal masses between January 2017 and March 2021 were analyzed. The Mann-Whitney U test or Kruskal-Wallis test was used for between-group comparisons of medians. Logistic regression was used to establish an EOC predictor model. Area under the curve (AUC) comparisons between models were performed using the Delong nonparametric approach.

**Results**: The median age of the patients was 43 years. Twenty-nine (43.9%) patients had early-stage disease (stages I-II) and 37 (56.1%) patients had advanced-stage disease (stages III-IV). The median age, body mass index, white blood cell count, hemoglobin-to-red cell distribution width ratio (HRR), platelet count, platelet-to-lymphocyte ratio, lymphocyte-to-monocyte ratio, serum albumin level, cancer antigen 125, human epididymal secretory protein 4 (HE4), ROMA, and CPH-I were significantly different between the stage I-IV EOC and benign ovarian mass groups. Multivariate logistic regression analysis revealed that HE4, HRR, and computed tomography (CT) imaging were significant predictors of both stages I-IV and I-II EOC. Using these covariates, an interim model (IM) (consisting of HE4 and HRR) and a full model (FM) (consisting of HE4, HRR, and CT imaging) were constructed. When predicting stage I-IV EOC, the AUC of IM was comparable to that of ROMA or CPH-I, whereas the AUC of FM outperformed ROMA or CPH-I. In predicting stage I-II EOC, the AUC of IM was comparable to that of CPH-I but higher than that of ROMA, and the AUC of FM outperformed ROMA or CPH-I.

**Conclusion**: FM outperformed ROMA or CPH-I in predicting stage I-IV EOC and stage I-II EOC. Therefore, FM could be a promising model for improving preoperative prediction of EOC at an early stage. However, further prospective studies are required to validate these results.

## Introduction

Epithelial ovarian cancer (EOC) has nonspecific symptoms and screening tests have not been shown to be effective. Consequently, EOC remains the leading cause of death in patients with gynecological malignancies 1. Therefore, the development of a valid model to predict the EOC at an early stage is required.

Cancer antigen 125 (CA125) and human epididymis secretory protein 4 (HE4) are key serum tumor markers for detecting ovarian malignancies. The area under the curve (AUC) of CA125 for predicting EOC ranged from 0.86 to 0.93 2-8. In a meta-analysis, the pooled sensitivity (SE) and specificity (SP) of serum CA125 for predicting EOC were 82% and 73%, respectively 2. HE4 is a key serum tumor marker used for the diagnosis of ovarian malignancies. HE4 is encoded by WAP four-disulfide core domain 2, which resides on the most frequently amplified genomic sequence in ovarian cancer 9. The AUC of HE4 in predicting EOC ranged from 0.87 to 0.94 2-8. In a meta-analysis, the pooled SE and SP of HE4 for predicting EOC were 73% and 90%, respectively 2.

The Risk of Ovarian Malignancy algorithm (ROMA), which is composed of CA125 and HE4, is a US Food and Drug Administration-cleared predictive algorithm. The AUC of ROMA in predicting EOC ranged from 0.90 to 0.95 2-6, 8, 10-13. In Moore et al.'s study, there was a significant difference in the AUC between ROMA and CA125 and no significant difference between ROMA and HE4 in predicting EOC 3. The Copenhagen Index (CPH-I) consists of age, CA125, and HE4. The AUC of CPH-I for predicting EOC ranged from 0.88 to 0.94 6, 10-12. The relative performance of CPH-I to ROMA for predicting stage I-IV EOC or stage I-II EOC is comparable 6, 10, 11. Although improvements have been made in the triage of women presenting with a pelvic mass by the addition of ROMA and CPH-I 14, further studies on possible predictors that can outperform ROMA or CPH-I are required.

Hematologic parameters such as hemoglobin (Hb) concentration are considered predictors of EOC 15. Red cell distribution width (RDW) predicts ovarian cancer (OC) with an SE and SP of 76.7% and 70.3%, respectively 16. Recently, the Hb-to-RDW ratio (HRR) has been reported to be a prognostic marker for malignant tumors, including head and neck cancer, lung cancer, esophageal cancer, gastric cancer, hepatoma, renal cell carcinoma, bladder cancer, and malignant lymphoma 17-21. Additionally, the diagnostic efficacy of HRR for breast, nasopharyngeal, and lung cancers has been reported 17, 22, 23. However, the clinical value of HRR in gynecological malignancies remains unknown. Considering the promising results of Hb and RDW as predictors of OC, the clinical value of HRR as a predictor of EOC may be more potent than a single test such as Hb and RDW. In addition, the lymphocyte-to-monocyte ratio (LMR) and platelet-to-lymphocyte ratio (PLR) have been reported to predict EOC 15. Therefore, the addition of hematologic parameters to serum tumor markers (e.g., CA 125 and HE4) could improve EOC prediction.

Computed tomography (CT) scans are more accurate than ultrasonography (USG) (94% vs. 80%) in differentiating malignant ovarian tumors (MOTs) from benign ovarian masses (BOMs) 24. Moreover, CT imaging provides an equally accurate diagnosis of MOTs with more reproducible image interpretation than USG 25, 26. In a meta-analysis, Wang et al. illustrated pooled SE and SP of 79% and 87%, respectively, of CT imaging for differentiating MOTs from BOMs 27. In addition, Midulla et al. illustrated that HE4, together with CT imaging, strengthens the clinical relevance of this study in the follow-up of patients with peritoneal carcinomatosis 28. Therefore, CT imaging combined with serum tumor markers can improve the preoperative detection of EOC.

In this study, we aimed to establish robust predictive models using multivariate logistic regression analysis for variables including demographic variables, hematologic parameters, CT imaging, and serum tumor markers (i.e., CA125 and HE4). We then compared the predictive power of the models with those of ROMA or CPH-I.

## Methods

### Patients

Consecutive patients with an adnexal mass who underwent imaging-guided biopsy, surgical biopsy, or surgical resection for definitive tissue diagnosis at Pusan National University Hospital between January 2017 and March 2021 were analyzed. The adnexal masses were histologically analyzed according to the 2014 World Health Organization classification of tumors of female reproductive organs.

The inclusion criteria were as follows: (i) EOCs or BOMs by histology; (ii) hematologic parameters including white blood cell count [WBC], erythrocyte parameters (Hb, mean corpuscular volume [MCV], and RDW), and platelet count; (iii) abdominopelvic CT imaging; and (iv) CA125 and HE4 levels. The exclusion criteria were as follows: (i) borderline ovarian tumors (BOTs), non-epithelial OC, and metastatic cancer to the ovary; (ii) synchronous malignancies; (iii) serum creatinine levels >1.5 mg/dL; and (iv) arterial oxygen saturation less than 90%.

This study was approved by the Institutional Review Board of the Pusan National University Hospital (IRB 2210-010-119). The requirement for informed consent was waived because of the retrospective design of the study.

### Baseline clinical characteristics

Data on age and body mass index (BMI) at the time of histological diagnosis were retrieved from patients' medical records. Menopausal status was classified into premenopausal (pre-M) and postmenopausal (post-M). Post-M women were those who had amenorrhea for more than one year or ≥50 years among those who had previously undergone hysterectomy.

The WBC count, absolute neutrophil count (ANC), absolute lymphocyte count (ALC), absolute monocyte count (AMC), Hb, MCV, RDW, platelet count, and serum albumin level (ALB) were performed within seven days before or on the day of tissue biopsy. If more than one test result was available, results close to the date of the tissue diagnosis were selected. The HRR was calculated by dividing Hb by RDW; the PLR by dividing the platelet count by the ALC; and the LMR by dividing the ALC by AMC.

Serum CA125 and HE4 levels, which were measured within 30 days before tissue diagnosis using a chemiluminescent enzyme immunoassay on a COBAS 6000 system (Roche, Switzerland), were retrieved 5. To calculate ROMA, the predictive index (PI) was calculated using CA125 and HE4 levels. The PI for pre-M women was calculated as follows: PI = -12.0 + 2.38 × ln (HE4) + 0.0626 × ln (CA125). The PI for post-M women was calculated as follows: PI = -8.09 + 1.04 × ln (HE4) + 0.732 × ln (CA125). ROMA was calculated as follows: ROMA = e^(PI)^/[1 + e^(PI)^] × 100 29, 30. CPH-I was calculated as follows: CPH-I = -14.0647 + 1.0649 × log2 (HE4) + 0.6050 × log2 (CA125) + 0.2672 × age/10. The predicted probability (PP) was calculated as follows: PP = e^(CPH-I)^/(1+e^(CPH-I)^) 6.

All patients had an adnexal mass documented on CT within 30 days before tissue diagnosis. CT examinations were performed using a 64-detector row CT scanner (Discovery 750 HD, Discovery CT 750 HD; GE Healthcare, Milwaukee, WI, USA) or 256-detector row CT scanner (Revolution; GE Healthcare, Milwaukee, WI, USA). CT scanning was initiated after intravenous administration of a nonionic iodinated contrast material. The CT findings were reported before surgical exploration by expert radiologists with more than five years of experience in female imaging.

### Statistical analysis

Continuous variables are reported as medians with interquartile ranges (IQR). Correlations between variables were determined using Pearson's correlation coefficient. The Mann-Whitney U test was used to determine the differences in the median values of the two groups for each variable. The Kruskal-Wallis test was used to determine the differences in the median values of more than two groups for each variable. The AUC comparisons between variables were performed using the Delong nonparametric approach.

Logistic regression analyses were performed on demographic variables, hematologic parameters, serum albumin level (ALB), serum tumor markers, and CT imaging. For continuous variables without validated cutoff points, dichotomization using an optimal cutoff point was not performed to avoid possible bias 31. The Hosmer-Lemeshow test was applied as a goodness-of-fit test for logistic regression analysis. Multicollinearity in the regression model was determined by calculating the variance inflation factors (VIFs) for the model covariates. Nomograms for predicting EOC were constructed using the optimal model and were internally validated using calibration curves. The AUCs of the established models were compared with those of ROMA or CPH-I.

Statistical analyses were performed using R package (R-project.org) and MedCalc® Statistical Software version 20.110 (MedCalc Software Ltd., Ostend, Belgium; https://www.medcalc.org, 2022). The two-sided significance level was set at 5% (*p* <0.05).

## Results

### Patients' clinical characteristics

Among the 1,047 patients initially assessed for eligibility, 327 without available CT images were excluded from the study. Among the 720 patients with available CT images, 55 were excluded for the following reasons: BOTs by histology (*n*=30), non-epithelial OC by histology (*n*=18), metastatic cancer to the ovary by histology (*n*=2), and serum creatinine level >1.5 mg/dL (*n*=5). Ultimately, 665 patients were included in this study.

The median patient age was 43 years (IQR: 31-55 years). Four hundred fifty-three (68.1%) patients were classified as pre-M, and 212 (31.9%) were classified as post-M. Serous carcinoma (*n*=42) was the most common EOC, followed by mucinous carcinoma (*n*=9), endometrioid carcinoma (*n*=8), and clear cell carcinoma (*n*=7). Twenty-nine (43.9%) patients had early-stage disease (stages I-II) and 37 (56.1%) patients had advanced-stage disease (stages III-IV). Endometriomas (*n*=195) were the most common BOMs, followed by mature cystic teratomas (*n*=170), mucinous cystadenomas (*n*=71), and serous cystadenomas (*n*=62) (Table 1).

### Pearson's correlation coefficients for variables in patients with EOC

**Figure 1:**
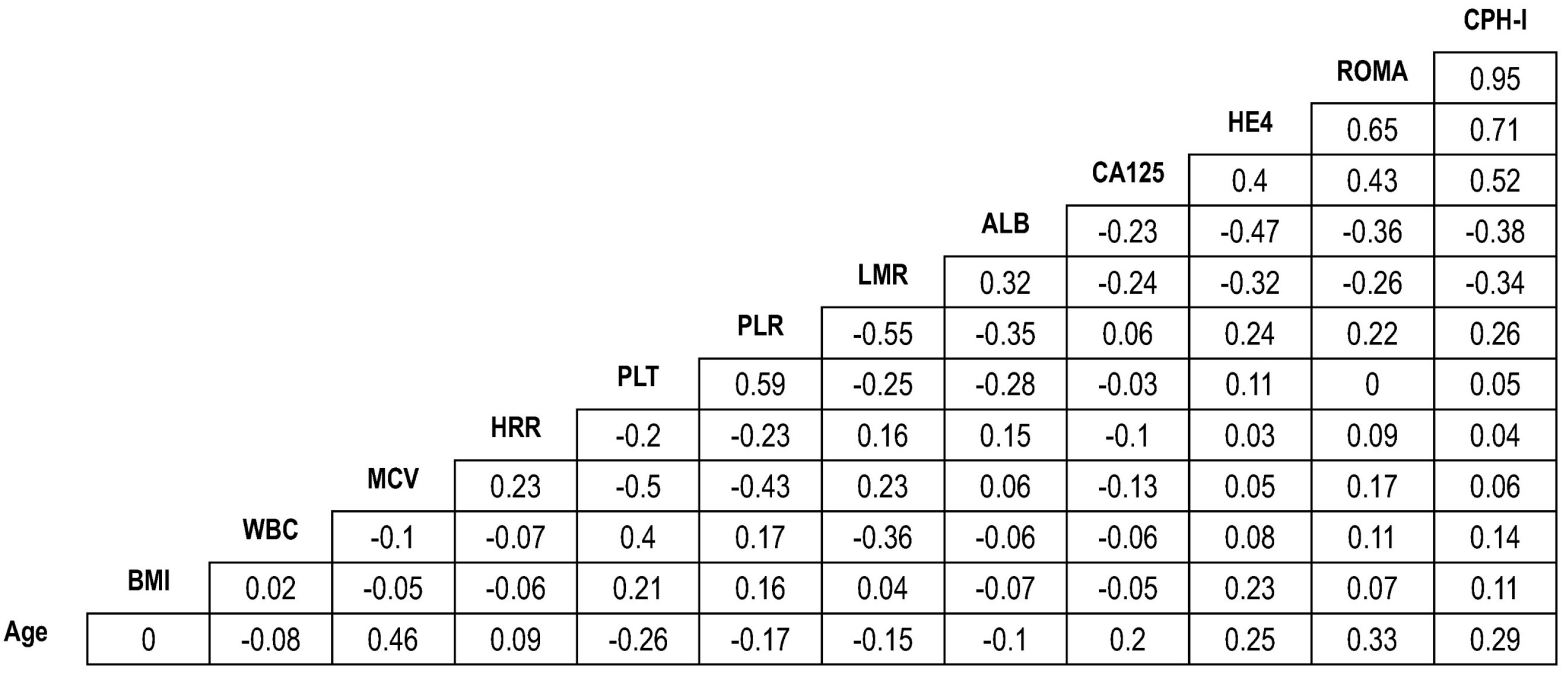
** Correlation coefficients of demographic and laboratory variables in patients with epithelial ovarian cancers.** The number in the box represents the correlation coefficient (*r*). ALB, albumin level; BMI, body mass index; CA125, cancer antigen 125; CPHI, Copenhagen index; HE4, human epididymis protein 4; HRR, hemoglobin-to-red cell distribution width ratio; LMR, lymphocyte-to-monocyte ratio; MCV, mean corpuscular volume; PLR, platelet-to-lymphocyte ratio; PLT, platelet count; ROMA, risk of ovarian malignancy algorithm; WBC, white blood cell.

Pearson's correlation coefficient illustrated significant correlations between ROMA and CPH-I (*r*=0.95), HE4 and ROMA (*r*=0.65), and HE4 and CPH-I (*r*=0.71). However, no significant correlations were observed between other pairs of variables (Fig. 1).

### Comparison of variable medians between EOC and BOM

The median values of age, BMI, WBC count, HRR, platelet count, PLR, LMR, ALB, CA125, HE4, ROMA, and CPH-I were significantly different between the stage I-IV EOC and BOM groups.

In the subgroup analyses, there were significant differences in the median values of age, HRR, PLR, LMR, ALB, CA125, HE4, ROMA, and CPH-I between the stage I-II EOC and BOM groups. Similarly, the median values of age, HRR, platelet count, PLR, LMR, ALB, CA125, HE4, ROMA, and CPH-I were significantly different between the stage III-IV EOC and BOM groups.

There were significant differences in the median values of PLR, ALB, CA125, HE4, ROMA, and CPH-I between EOC stages I-II and III-IV (Table 2).

### AUC Comparison of EOC Predictors

**Figure 2:**
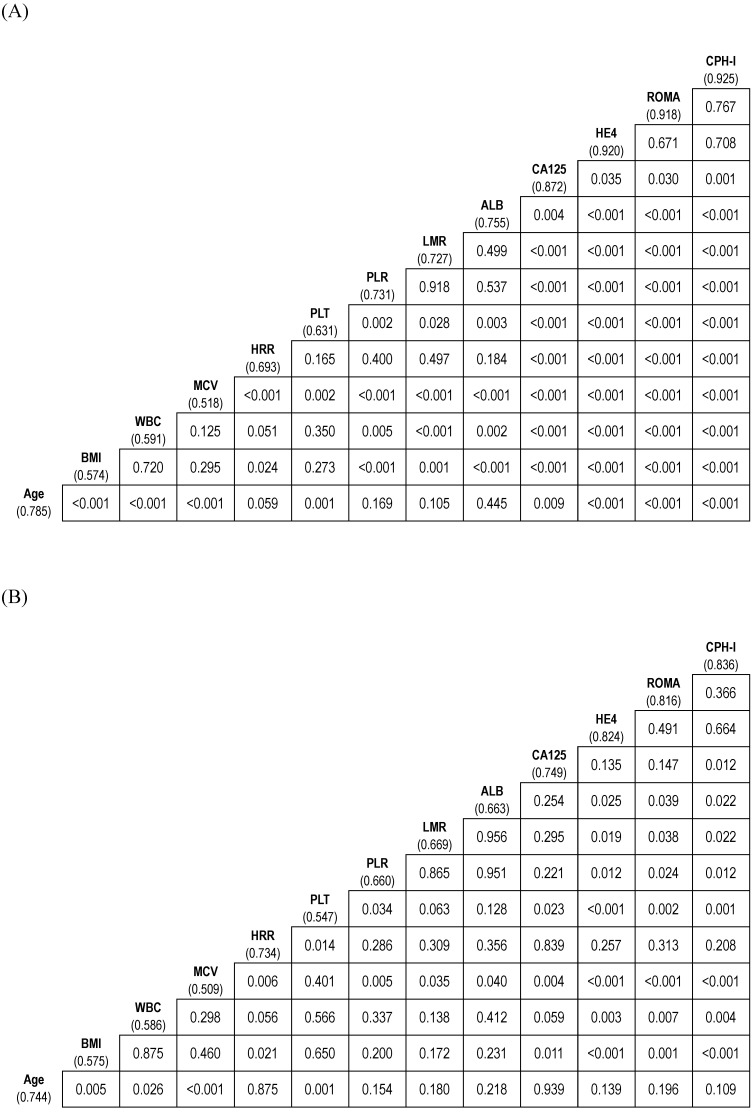
** Comparison of area under the curves of variables for differentiation of epithelial ovarian cancers from benign ovarian masses. (A)** Stage I-IV epithelial ovarian cancer; **(B)** Stage I-II epithelial ovarian cancer. The numbers in parentheses represent the area under the curve. The numbers in the box represent *p*-values. ALB, albumin level; BMI, body mass index; CA125, cancer antigen 125; CPH-I, Copenhagen index; HE4, human epididymis protein 4; HRR, hemoglobin-to-red cell distribution width ratio; LMR, lymphocyte-to-monocyte ratio; MCV, mean corpuscular volume; PLR, platelet-to-lymphocyte ratio; PLT, platelet count; ROMA, risk of ovarian malignancy algorithm; WBC, white blood cell.

The AUCs of the variables for predicting stage I-IV EOC were age (0.785), BMI (0.574), WBC (0.591), MCV (0.518), HRR (0.693), platelet count (0.631), PLR (0.731), LMR (0.727), ALB (0.755), CA125 (0.872), HE4 (0.920), ROMA (0.918), and CPH-I (0.925). The AUC of HRR was lower than those of CA125, HE4, ROMA, or CPH-I. Similarly, the AUC of CA125 was lower than those of HE4, ROMA, and CPH-I. No significant differences in AUCs were observed between HE4, ROMA, and CPH-I (Fig. 2A).

The AUCs of the variables for predicting stage I-II EOC were as follows: age (0.744); BMI (0.575); WBC (0.586); MCV (0.509); HRR (0.734); platelet count (0.547); PLR (0.660); LMR (0.669); ALB (0.663); CA125 (0.749); HE4 (0.824); ROMA (0.816); and CPH-I (0.836). No significant differences in AUCs were observed between the HRR and CA125, HE4, ROMA, or CPH-I levels. The AUC of CA125 was comparable to that of HE4 or ROMA but lower than that of CPH-I. No significant differences in AUCs were observed between HE4, ROMA, and CPH-I (Fig. 2B).

### Logistic regression analysis of variables for predicting EOC

The significant variables for predicting stage I-IV EOC by univariate analysis were age (odds ratio [OR] 1.07; *p*<0.001), WBC (OR 1.00; *p*=0.017), HRR (OR 0.02; *p*<0.001), platelet count (OR 1.00; *p*<0.001), PLR (OR 1.01; *p*<0.001), LMR (OR 0.60; *p*<0.001), ALB (OR 0.10; *p*<0.001), CA125 (OR 1.01; *p*<0.001), HE4 (OR 1.05; *p*<0.001), and CT imaging (OR 93.02; *p*<0.001). Multivariate analysis revealed that HE4 (odds ratio [OR] 1.04; *p<*0.001), HRR (OR 0.02; *p<*0.001), and CT imaging (OR 27.27; *p<*0.001) were significant variables. A good fit for the logistic regression model was found using the Hosmer-Lemeshow test. The VIF values for HE4, HRR, and CT imaging were 1.40, 1.07, and 1.44, respectively, indicating no significant multicollinearity in the regression model (Table 3).

The significant variables for predicting stage I-II EOC by univariate analysis were as follows: age (OR 1.06; *p*<0.001), HRR (OR 0.01; *p*<0.001), platelet count (OR 1.00; *p*=0.020), PLR (OR 1.01; *p*<0.001), LMR (OR 0.70; *p*=0.003), ALB (OR 0.21; *p*<0.001), CA125 (OR 1.00; *p*<0.001), HE4 (OR 1.04; *p*<0.001), and CT imaging (OR 55.65; *p*<0.001). Multivariate analysis revealed that HE4 (OR 1.04; *p<*0.001), HRR (OR 0.02; *p<*0.001), and CT imaging (OR 27.48; *p<*0.001) were significant variables. A good fit for the logistic regression model was found using the Hosmer-Lemeshow test. The VIF values for HE4, HRR, and CT were 1.11, 1.05, and 1.14, respectively, indicating no significant multicollinearity in the regression model (Table 3).

### Model setup for EOC prediction

**Figure 3:**
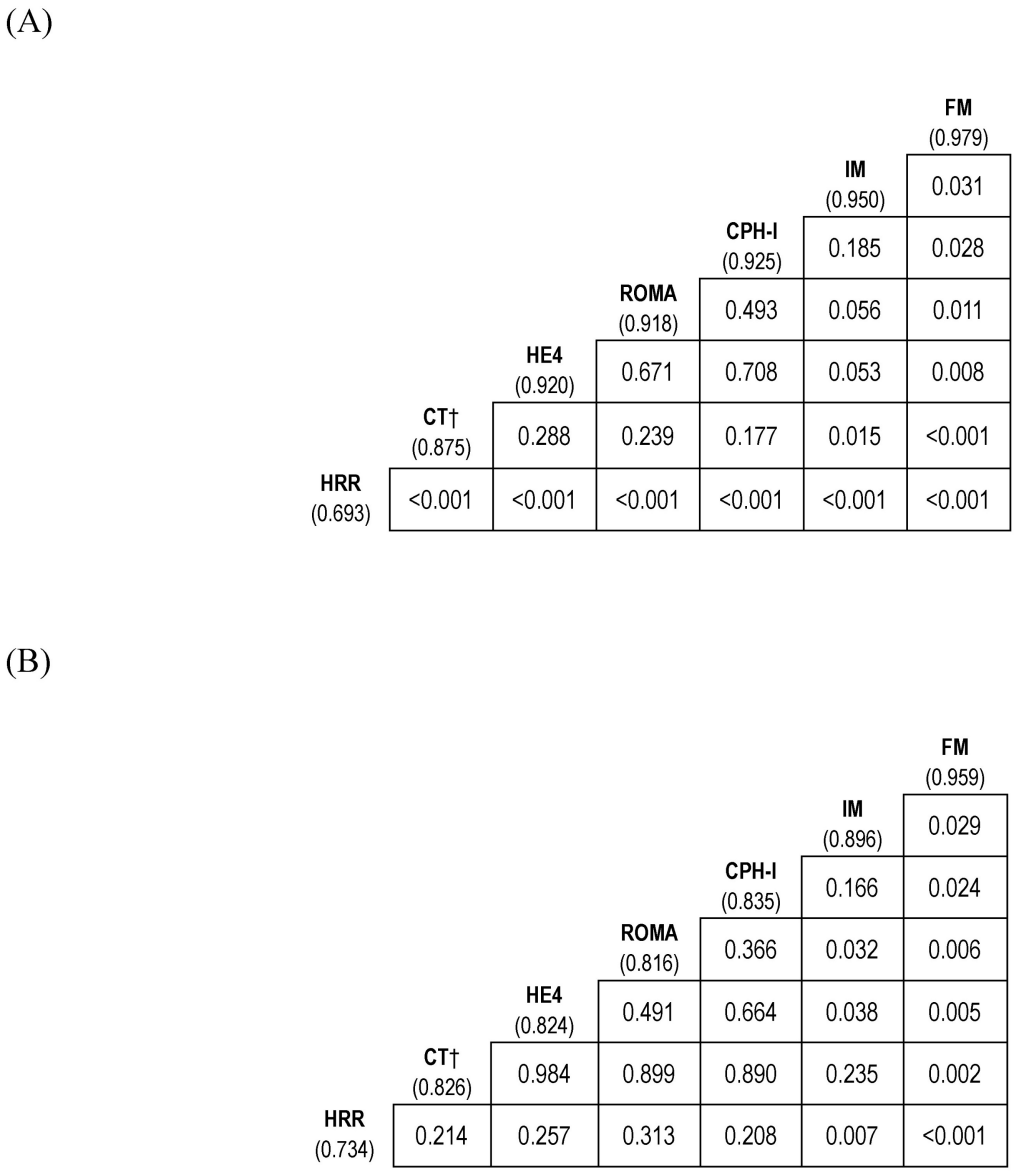
** Comparison of diagnostic performance of biomarker combinations for differentiation of epithelial ovarian cancers from benign ovarian masses. (A)** Stage I-IV epithelial ovarian cancer; **(B)** Stage I-II epithelial ovarian cancer. † Categorical variable. The numbers in parentheses represent the area under the curve. The numbers in the box represent *p*-values. CPH-I, Copenhagen index; CT, computed tomography; FM, full model (consisting of HE4, HRR, and CT imaging); HE4, human epididymis protein 4; HRR, hemoglobin-to-red cell distribution width ratio; IM, interim model (consisting of HE4 and HRR); ROMA, risk of ovarian malignancy algorithm.

On constituting the interim model (IM) (consisting of HE4 and HRR) and full model (FM) (consisting of HE4, HRR, and CT imaging) for predicting stage I-IV EOC, the AUC values of the IM and FM were 0.950 and 0.979, respectively. The FM had a higher AUC than that of the IM (*p=*0.031) (Fig. 3A).

When constituting the predictor models (IM and FM) for predicting stage I-II EOC, the AUCs for IM and FM were 0.896 and 0.959, respectively. The AUC of FM was higher than that of IM (*p=*0.029) (Fig. 3B).

**Figure 4:**
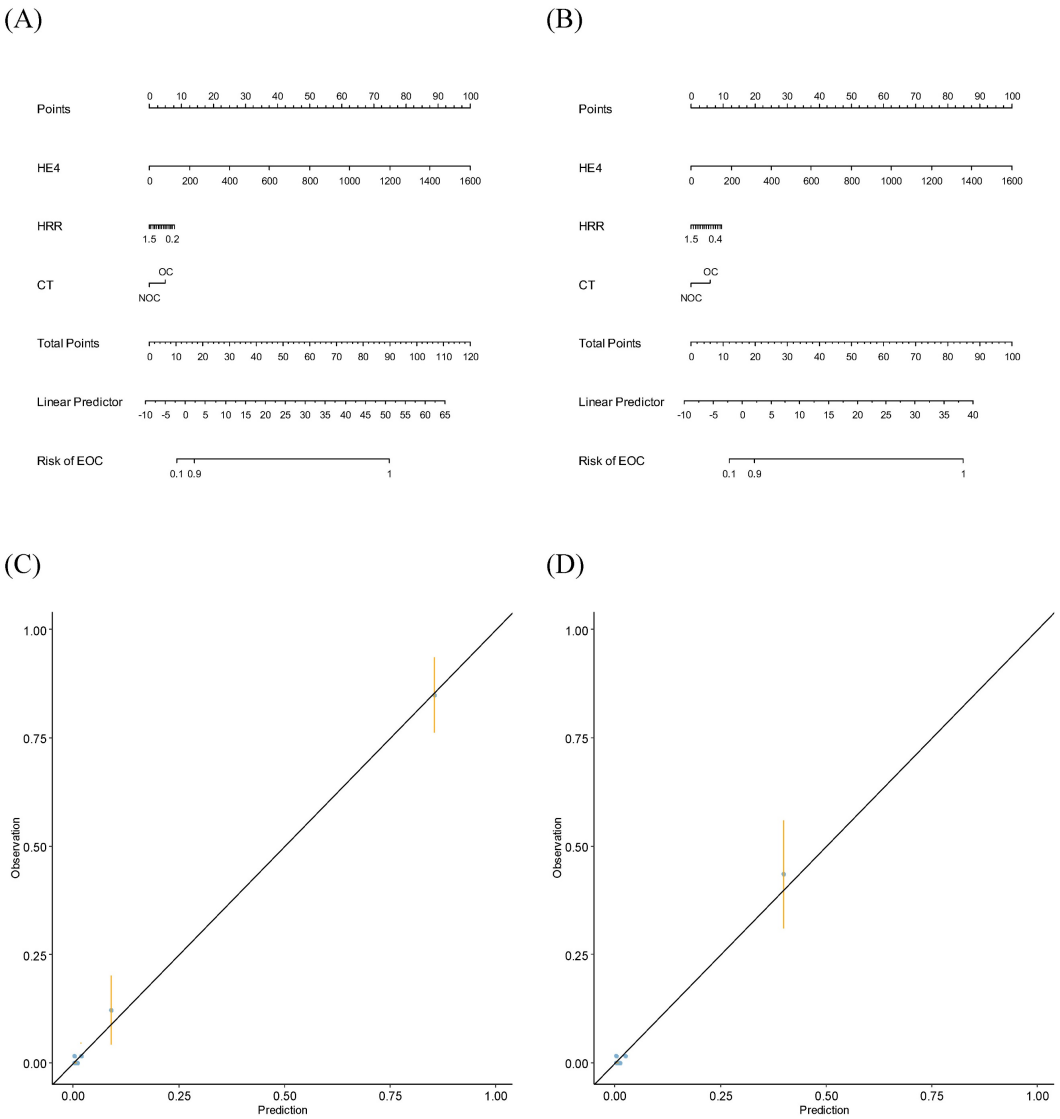
** Nomograms and calibration curves predicting epithelial ovarian cancers. (A)** Nomogram predicting stage I-IV EOC; **(B)** Nomogram predicting stage I-II EOC; **(C)** Calibration curve predicting stage I-IV EOC; **(D)** Calibration curve predicting stage I-II EOC.CT, computed tomography; EOC, epithelial ovarian cancer; HE4, human epididymis protein 4; HRR, hemoglobin-to-red cell distribution width ratio; NOC, no ovarian cancer; OC, ovarian cancer.

Using FM, nomograms were established to predict stage I-IV or stage I-II EOC (Fig. 4A and Fig. 4B). When validating the nomograms using calibration curves, the predictions closely matched the actual observations (Fig. 4C and Fig. 4D).

### Comparison of models for EOC prediction with ROMA or CPH-I

**Figure 5:**
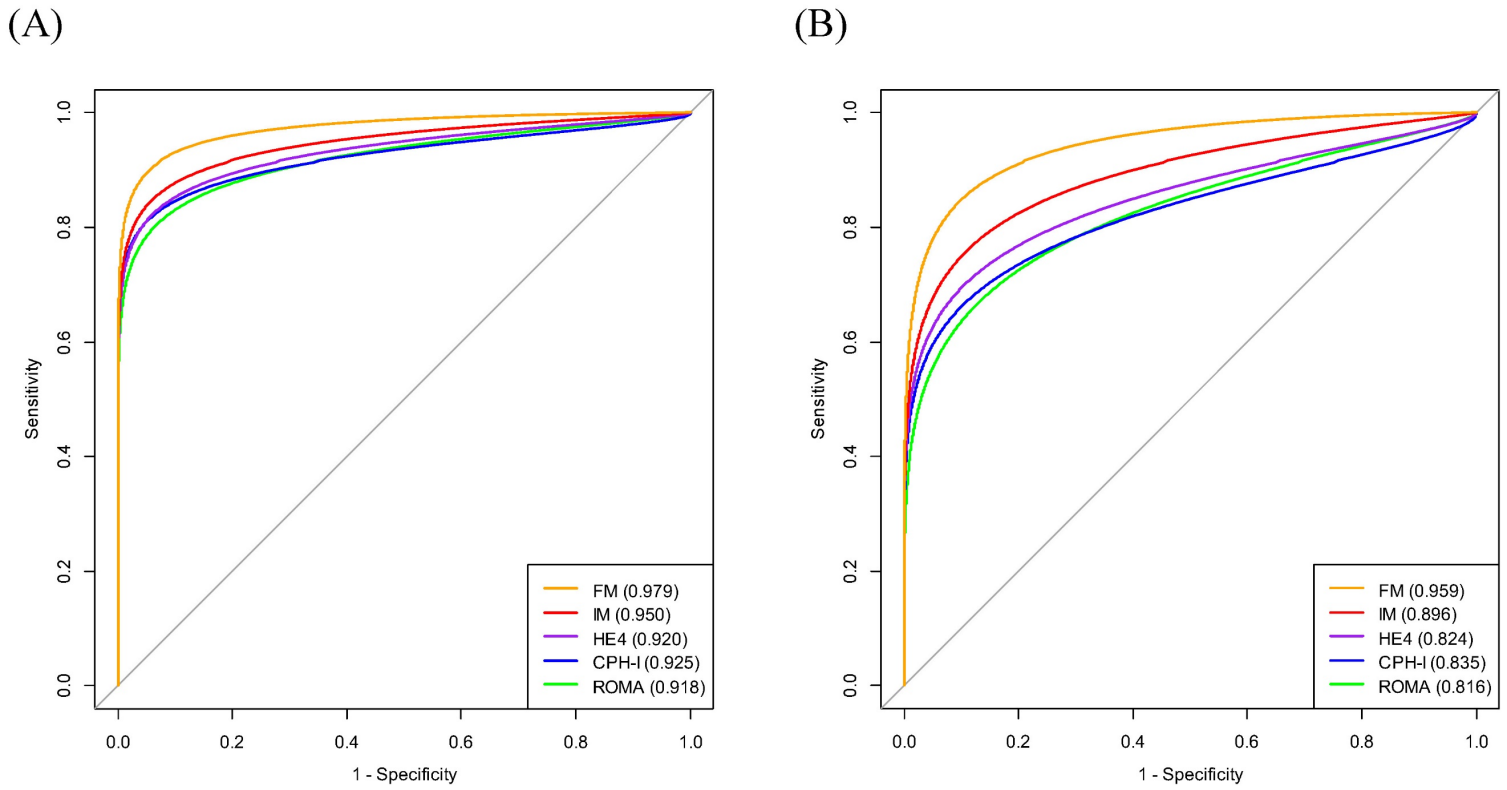
** Comparison of diagnostic performance of biomarker combinations for differentiation of epithelial ovarian cancers from benign ovarian masses. (A)** Stage I-IV epithelial ovarian cancer; **(B)** Stage I-II epithelial ovarian cancer. The numbers in parentheses represent the area under the curve. CPH-I, Copenhagen index; CT, computed tomography; FM, full model (consisting of HE4, HRR, and CT imaging); HE4, human epididymis protein 4; HRR, hemoglobin-to-red cell distribution width ratio; IM, interim model (consisting of HE4 and HRR); ROMA, risk of ovarian malignancy algorithm.

The AUC of FM for predicting stage I-IV EOC outperformed that of ROMA or CPH-I (*p*=0.011 and *p*=0.028, respectively). Whereas the AUC of IM was comparable to that of ROMA or CPH-I (Fig. 3A and Fig. 5A).

The AUC of FM for predicting stage I-II EOC outperformed that of ROMA or CPH-I (*p*=0.006 and *p*=0.024, respectively). Although the AUC of IM was comparable to that of CPH-I, it was higher than that of ROMA (*p*=0.032) (Fig. 3B and Fig. 5B).

## Discussion

EOC is the leading cause of death in patients with gynecological malignancies, as screening tests for EOC have not been shown to be effective. Therefore, the development of a valid predictor of EOC at an early stage (stages I-II) is necessary. In the present study, HE4, HRR, and CT imaging were significant predicters of stage I-IV or stage I-II EOC using multivariate logistic regression analysis.

HE4 is a key serum tumor marker used for the diagnosis of ovarian malignancies. HE4 overexpression reduces the activation of cytotoxic T cells and natural killer cells and increases the expression of programmed cell death ligand 1 in both tumor cells and macrophages 9. HE4 is encoded by WAP four-disulfide core domain 2, which resides on the most frequently amplified genomic sequence in OC 9. In this study, the AUC of HE4 for predicting stage I-IV EOC was 0.920, and the results were consistent with those of previous studies 2-8. However, caution should be exercised because HE4 levels may increase in older adults, smokers, and in those with renal disease, lung cancer, and endometrial cancer 32. Therefore, in the present study, HE4 was adjusted for age in multivariate analysis, and patients with serum creatinine level >1.5 mg/dL, and concurrent malignant tumors were excluded from the study.

The HRR is a prognostic marker for various malignant tumors 17-21. In a meta-analysis by Chi et al., a low HRR was associated with an increased risk of all-cause mortality (hazard ratio [HR] 2.29) and disease progression or relapse (HR 2.19). Additionally, the diagnostic efficacy of HRR for breast, nasopharyngeal, and lung cancers has been reported 17, 22, 23. However, the diagnostic efficacy of HRR for OCs has not been reported. In the present study, HRR was an independent predictor of both stage I-IV and I-II EOC in multivariate logistic regression analysis. Additionally, there were significant differences in median values between BOM and stage I-II EOC, and between BOM and stage I-IV EOC groups, illustrating the diagnostic efficacy of HRR even in early stage EOC. Although the mechanisms underlying the clinical significance of HRR are unclear, HRR combines the effect of both Hb and RDW, which would provide more diagnostic efficacy than a single variable (Hb or RDW) in predicting OC 15, 16, 19. Low Hb levels (i.e., anemia) are related to impaired nutrition and immune system status and are considered to predict poor outcomes in various malignant tumors 33. Additionally, low Hb concentration is considered a predictor of EOC 15. RDW is the variability in the MCV of red blood cells and predicts survival outcomes in various malignant tumors 33. RDW also predicts OC, with SE and SP values of 76.7% and 70.3%, respectively 16. Despite these observations, the exact mechanism of RDW remains unclear, but it is speculated to be driven by neoplastic-driven inflammatory processes 34.

CT imaging has advantages, including excellent spatial resolution, and appears to be more accurate than USG (94% vs. 80%) in differentiating malignant ovarian tumors (MOTs) from BOMs 24. This imaging modality also provides an equally accurate diagnosis of MOTs with more reproducible image interpretation than USG 25, 26. However, early stage (stage I-II) OC and peritoneal carcinosis with implants ≤1 cm in maximum diameter are difficult to identify on CT 35, 36. In the present study, CT imaging was a significant variable in the multivariate logistic regression analysis. The SE and SP of CT imaging for differentiating stage I-IV EOC from BOM were 78.8% and 96.2%, respectively. The results of the present study are somewhat better than those of Wang et al., who showed that the pooled SE and SP of CT imaging for differentiating MOTs from BOM were 79% and 87%, respectively 27. However, the inclusion of non-epithelial OC, metastatic cancer to the ovary, and BOTs in addition to EOC in a previous study may have attenuated the predictive power of CT imaging.

Following the establishment of predictor models, their AUC values were compared with those of ROMA or CPH-I. In this study, the AUC of ROMA in predicting stage I-IV EOC was 0.918; the results were consistent with those of previous studies 2-6, 8, 10-13, 37. Furthermore, the AUC of ROMA in predicting stage I-II EOC was 0.816; these results were consistent with those of previous studies 6, 10, 11. In the present study, the AUC of CPH-I for predicting stage I-IV EOC was 0.925. The results of the present study are consistent with those of previous studies 6, 10-12. Additionally, the AUC of CPH-I in predicting stage I-II EOC was 0.835; these results were consistent with those of previous studies 6, 10, 11. ROMA and CPH-I were significantly correlated (*r*=0.95), as previously reported 6, 10-12. Moreover, no significant differences in AUCs were found between ROMA and CPH-I in predicting stages I-IV and I-II EOC.

When comparing IM with ROMA or CPH-I, the AUC of IM was comparable to that of ROMA or CPH-I for predicting stage I-IV EOC. However, IM had a higher AUC than ROMA (*p=*0.032) for predicting stage I-II EOC. On comparing FM with ROMA or CPH-I, the AUC of FM was significantly higher than that of ROMA or CPH-I in predicting both stages I-IV and I-II EOC.

This study has the following strengths. First, to the best of our knowledge, this is the first study on the clinical value of HRR in patients with gynecological malignancies. The median HRR values in stages I-IV and I-II EOC were lower than those of the BOMs. Furthermore, HRR was a significant predictor of both stage I-IV and I-II EOC in multivariate logistic regression analysis. The use of HRR in the model has the advantage of obtaining results while examining complete blood counts and avoids additional costs. Second, the results of the multivariate analysis revealed that HRR, HE4, and CT imaging were independent predictors of both stages I-IV and I-II EOC. On constituting the predictor models in predicting stage I-IV EOC, the AUCs of the IM and FM were 0.950 and 0.979, respectively. Additionally, when constituting the predictor models for stage I-II EOC, the AUCs of IM and FM were 0.896 and 0.959, respectively. Third, the AUC of FM was significantly higher than that of ROMA or CPH-I in predicting stages I-IV and I-II EOC. In addition, the AUC of IM was comparable to that of ROMA or CPH-I for stage I-IV EOC predictions, but higher than ROMA for stage I-II EOC predictions.

The limitations of the present study include its retrospective nature and recruitment from a single institution. In addition, CT data were not available for 327 (31.2%) of the 1047 patients who were initially assessed for eligibility. Therefore, the results of this study may have been affected by these factors. Nevertheless, we included several consecutive patients from the same teaching hospital, which allowed us to achieve a high degree of uniformity in the procedure.

In conclusion, FM consisting of HE4, HRR, and CT imaging outperformed ROMA or CPH-I in predicting stage I-IV EOC. Moreover, FM outperformed ROMA or CPH-I in predicting stage I-II EOC. Therefore, FM could be a promising model for improving preoperative prediction of EOC at an early stage. However, further prospective studies are required to validate these results.

## Data Access Statement

The datasets generated and/or analyzed during the current study are available from the corresponding author upon reasonable request.

## Ethical Compliance

All procedures performed in this study involving human participants were in accordance with the ethical standards of the institutional and/or national research committee, and the 1964 Declaration of Helsinki and its later amendments or comparable ethical standards.

## Author Contributions

According to the recommendation of ICMJE, Tae-Kyu Jang, Heungyeol Kim, Wankyu Eo, Ki Hyung Kim, Chul Min Lee, and Minyoung Kim contributed as authors.

Tae-Kyu Jang, Heungyeol Kim, Wankyu Eo, Ki Hyung Kim, Chul Min Lee, and Minyoung Kim contributed to the conception or design of the work.

Tae-Kyu Jang, Heungyeol Kim, Wankyu Eo, Ki Hyung Kim, Chul Min Lee, and Minyoung Kim contributed the acquisition, analysis, or interpretation of data for the work.

Tae-Kyu Jang, Heungyeol Kim, Wankyu Eo, Ki Hyung Kim, Chul Min Lee, and Minyoung Kim drafted the work or critically revised it for important intellectual content, finally approved the version to be published, and agreed to be accountable for all aspects of the work in ensuring that questions related to the accuracy or integrity of any part of the work are appropriately investigated and resolved.

## Figures and Tables

**Table 1:** Histopathologic types of patients with adnexal masses.

Classification	Histology
Malignant	
Epithelial ovarian cancer (*n*=66)	Serous carcinoma (*n*=42)
	Mucinous carcinoma (*n*=9)
	Endometrioid carcinoma (*n*=8)
	Clear cell carcinoma (*n*=7)
Benign	
Epithelial ovarian tumor (*n*=150)	Mucinous cystadenoma (*n*=71)
	Serous cystadenoma (*n*=62)
	Seromucinous cystadenoma (*n*=9)
	Others (*n*=8)
Germ cell tumor (*n*=170)	Mature cystic teratoma (*n*=170)
Sex cord-stromal tumor (*n*=24)	Fibroma/fibrothecoma (*n*=24)
Non-neoplastic mass (*n*=255)	Endometrioma (*n*=195)
	Paratubal cyst/paraovarian cyst (*n*=25)
	Simple or follicular cyst (*n*=18)
	Others (*n*=17)

**Table 2:** Comparison of median values between epithelial ovarian cancers and benign ovarian masses.

Variables	Median (IQR)				*p*-value†
	BOM (*n*=599)	Stage I-II EOC (*n*=29)	Stage III-IV EOC (*n*=37)	Stage I-IV EOC (*n*=66)	
Age (years)	41.0 (30.0-52.0)	54.0 (45.0-64.0)^a^	57.0 (52.0-68.0)^a^	56.0 (48.0-66.0)^a^	<0.001
BMI (kg/m^2^)	23.0 (21.0-25.0)	24.0 (22.0-26.0)	23.0 (21.0-26.0)	23.5 (21.0-26.0)^a^	0.138
WBC (per µL)	5990 (5060-7345)	6630 (5180-8980)	6650 (5420-8040)	6640 (5390-8610)^a^	0.052
MCV (fL)	90.9 (87.6-93.5)	90.6 (88.0-93.5)	90.7 (87.8-92.5)	90.7 (87.9-92.6)	0.878
HRR	1.008 (0.932-1.078)	0.898 (0.303-0.987)^a^	0.879 (0.777-1.035)^a^	0.888 (0.331-1.024)^a^	<0.001
Platelet ( 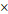 10^3^/µL)	270 (228-317)	272 (239-355)	331 (261-380)^a^	306 (249-379)^a^	<0.001
PLR	139.0 (113.7-171.2)	160.8 (134.8-215.8)^a^	220.1 (171.9-281.0)^ab^	192.6 (149.4-255.5)^a^	<0.001
LMR	5.2 (4.0-6.5)	4.1 (3.3-5.2)^a^	3.3 ( 2.3-4.5)^a^	3.6 (2.7-5.0)^a^	<0.001
Albumin (g/dL)	4.7 (4.5-4.9)	4.5 (4.1-4.8)^a^	4.1 ( 3.8-4.5)^ab^	4.4 (3.9-4.6)^a^	<0.001
CA125 (U/mL)	17.8 (10.9-35.9)	88.2 (20.7-317.0)^a^	444.0 (248.0-1500.0)^ab^	278.0 (64.7-777.0)^a^	<0.001
HE4 (pmol/L)	46.5 (40.8-55.2)	92.3 (50.4-188.0)^a^	431.9 (209.0-654.0)^ab^	215.3 (92.3-459.0)^a^	<0.001
ROMA (%)	7.0 (5.0-10.7)	38.2 (9.0-79.3)^a^	95.6 (79.2-98.0)^ab^	80.5 (34.2-97.1)^a^	<0.001
CPH-I (%)	1.2 (0.7-2.5)	15.9 (5.1-51.3)^a^	91.4 (60.8-98.2)^ab^	61.5 (12.0-96.2)^a^	<0.001

† Between BOM, stage I-II EOC, and stage III-IV EOC; a, *p*<0.05, compared with BOM; b, *p*<0.05, compared with stage I-II EOC. BMI, body mass index; BOM, benign ovarian mass; CA125, cancer antigen 125; CPH-I, Copenhagen index; EOC, epithelial ovarian cancer; HE4, human epididymis protein 4; HRR, hemoglobin-to-red cell distribution width ratio; IQR, interquartile range; LMR, lymphocyte-to-monocyte ratio; MCV, mean corpuscular volume; PLR, platelet-to-lymphocyte ratio; ROMA, risk of ovarian malignancy algorithm; WBC, white blood cell.

**Table 3:** Logistic regression analyses.

	Univariate		Multivariate	
Variables	OR (95% CI)	*p*-value	OR (95% CI)	*p*-value
**Stage I**-**IV EOC**				
Age (years)	1.07 (1.05-1.09)	<0.001		
BMI (kg/m^2^)	1.03 (0.98-1.09)	0.236		
WBC (per µL)	1.00 (1.00-1.00)	0.017		
MCV (fL)	0.99 (0.95-1.04)	0.732		
HRR	0.02 (0.01-0.05)	<0.001	0.02 (0.00-0.19)	<0.001
Platelet ( 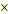 10^3^/µL)	1.00 (1.00-1.00)	<0.001		
PLR	1.01 (1.01-1.01)	<0.001		
LMR	0.60 (0.51-0.71)	<0.001		
Albumin (g/dL)	0.10 (0.05-0.18)	<0.001		
CA125 (U/mL)	1.01 (1.00-1.01)	<0.001		
HE4 (pmol/L)	1.05 (1.04-1.06)	<0.001	1.04 (1.03-1.06)	<0.001
CT (OC vs. NOC)	93.02 (45.17-191.57)	<0.001	27.27 (9.34-79.55)	<0.001
**Stage I**-**II EOC**				
Age (years)	1.06 (1.03-1.08)	<0.001		
BMI (kg/m^2^)	1.03 (0.95-1.11)	0.471		
WBC (per µL)	1.00 (1.00-1.00)	0.074		
MCV (fL)	0.98 (0.92-1.04)	0.428		
HRR	0.01 (0.00-0.03)	<0.001	0.02 (0.00-0.20)	<0.001
Platelet (×10^3^/µL)	1.00 (1.00-1.00)	0.020		
PLR	1.01 (1.00-1.01)	<0.001		
LMR	0.70 (0.56-0.88)	0.003		
Albumin (g/dL)	0.21 (0.10-0.46)	<0.001		
CA125 (U/mL)	1.00 (1.00-1.01)	<0.001		
HE4 (pmol/L)	1.04 (1.03-1.05)	<0.001	1.04 (1.02-1.05)	<0.001
CT (OC vs. NOC)	55.65 (22.85-135.56)	<0.001	27.48 (8.67-87.10)	<0.001

CA125, cancer antigen 125; BMI, body mass index; CI, confidence interval; CT, computed tomography; EOC, epithelial ovarian cancer; HE4, human epididymis protein 4; HRR, hemoglobin-to-red cell distribution width ratio; LMR, lymphocyte-to-monocyte ratio; MCV, mean corpuscular volume; NOC, no ovarian cancer; OC, ovarian cancer; OR, odds ratio; PLR, platelet-to-lymphocyte ratio.

## Abbreviations

ALB: albumin
AUC: area under the curve
BMI: body mass index
BOT: borderline ovarian tumor
CA125: cancer antigen 125
CPH-I: Copenhagen index
CT: computed tomography
EOC: epithelial ovarian cancer
Hb: hemoglobin
HE4: human epididymis secretory protein 4
HRR: hemoglobin-to-red cell distribution width ratio
IQR: interquartile range
LMR: lymphocyte-to-monocyte ratio
OC: ovarian cancer
OR: odds ratio
PI: predictive index
PLR: platelet-to-lymphocyte ratio
post-M: postmenopausal
PP: predicted probability
pre-M: premenopausal
RDW: red cell distribution width
ROC: receiver operating characteristic
ROMA: risk of ovarian malignancy algorithm
SE: sensitivity
SP: specificity
USG: ultrasonography
VIF: variance inflation factor
WBC: white blood cell
